# Meningitis retention syndrome caused by varicella zoster virus in a patient without a rash: a case report

**DOI:** 10.1186/s12879-021-06692-6

**Published:** 2021-09-23

**Authors:** Tsuneaki Kenzaka, Ken Goda, Ayako Kumabe

**Affiliations:** 1 Department of Internal Medicine, Hyogo Prefectural Tamba Medical Center, Tanba, Japan; 2 Division of Community Medicine and Career Development, Kobe University Graduate School of Medicine, 2-1-5, Arata-cho, Hyogo-ku, Kobe, Hyogo 652-0032 Japan

**Keywords:** Meningitis retention syndrome, Varicella zoster virus, Zoster sine herpete, Acute urinary retention, Lower limb dysesthesia

## Abstract

**Background:**

Meningitis retention syndrome (MRS) is a rare condition that presents with acute urinary retention as a complication of aseptic meningitis. Cases of MRS due to varicella zoster virus (VZV) infection without a rash are rare. We report the case of a patient who had no signs of meningitis or VZV infection, including a rash.

**Case presentation:**

A 58-year-old man presented with dysesthesia of the lower limbs and acute urinary retention. He had fever but no rash and no signs of meningitis. He was diagnosed to have VZV infection based on the detection of VZV DNA in the cerebrospinal fluid. He responded satisfactorily to a course of intravenous acyclovir and experienced no sequelae during a 2-year follow-up period.

**Conclusion:**

MRS due to aseptic meningitis of viral origin should be considered in the differential diagnosis of acute urinary retention even in the absence of specific signs and symptoms of meningitis or a suggestive rash.

## Background

The usual causes of acute urinary retention in adults include prostatic hyperplasia, peripheral nerve diseases involving the sacral spinal cord, such as diabetic neuropathy and Guillain-Barré syndrome, and diseases of the lumbar spinal canal, such as lumbar spondylosis and lumbar disc herniation. However, acute urinary retention has also been reported in association with cerebral demyelinating diseases such as acute disseminated encephalomyelitis and multiple sclerosis, aseptic meningitis, and herpes in the sacral region [1]. Meningitis retention syndrome (MRS) is a rare condition associated with aseptic meningitis presenting with acute urinary retention [2]. Enteroviruses, flaviviruses, arboviruses, and herpes simplex viruses are common viral causes of MRS, whereas varicella zoster virus (VZV) is rarely associated with MRS [4, 5].

A typical skin rash is not observed in about 40% of patients with meningoencephalitis caused by VZV [6]. However, besides fever, most patients with aseptic meningitis, including VZV meningitis, report symptoms such as headache, nausea and vomiting, signs of meningeal irritation, disturbance of consciousness, and brain dysfunction (cognitive dysfunction, behavioral changes, neurological symptoms, and convulsions) [7–9].

Here, we report about a patient with MRS associated with aseptic meningitis due to VZV who had no typical symptoms of meningitis or VZV infection.

## Case presentation

A 58-year-old man with no particular past medical history had fever of 38 °C 11 days before admission. Dysesthesia of both the lower limbs and acute urinary retention were observed in the same day. Although the fever improved after a few days, 7 days before admission, the patient developed urinary retention 7 days before admission, which required intervention by a urologist. As urinary drainage and oral administration of silodosin, urapidil, baclofen, and distigmine did not improve the patient’s condition, however, a spinal cord lesion was suspected and he was referred to our institution.

On systemic review during a medical examination at our institution, bilateral lower limb dysesthesia, dysuria, and constipation were present, whereas fever, headache, nausea, vomiting, and rash were absent.

**Fig. 1:**
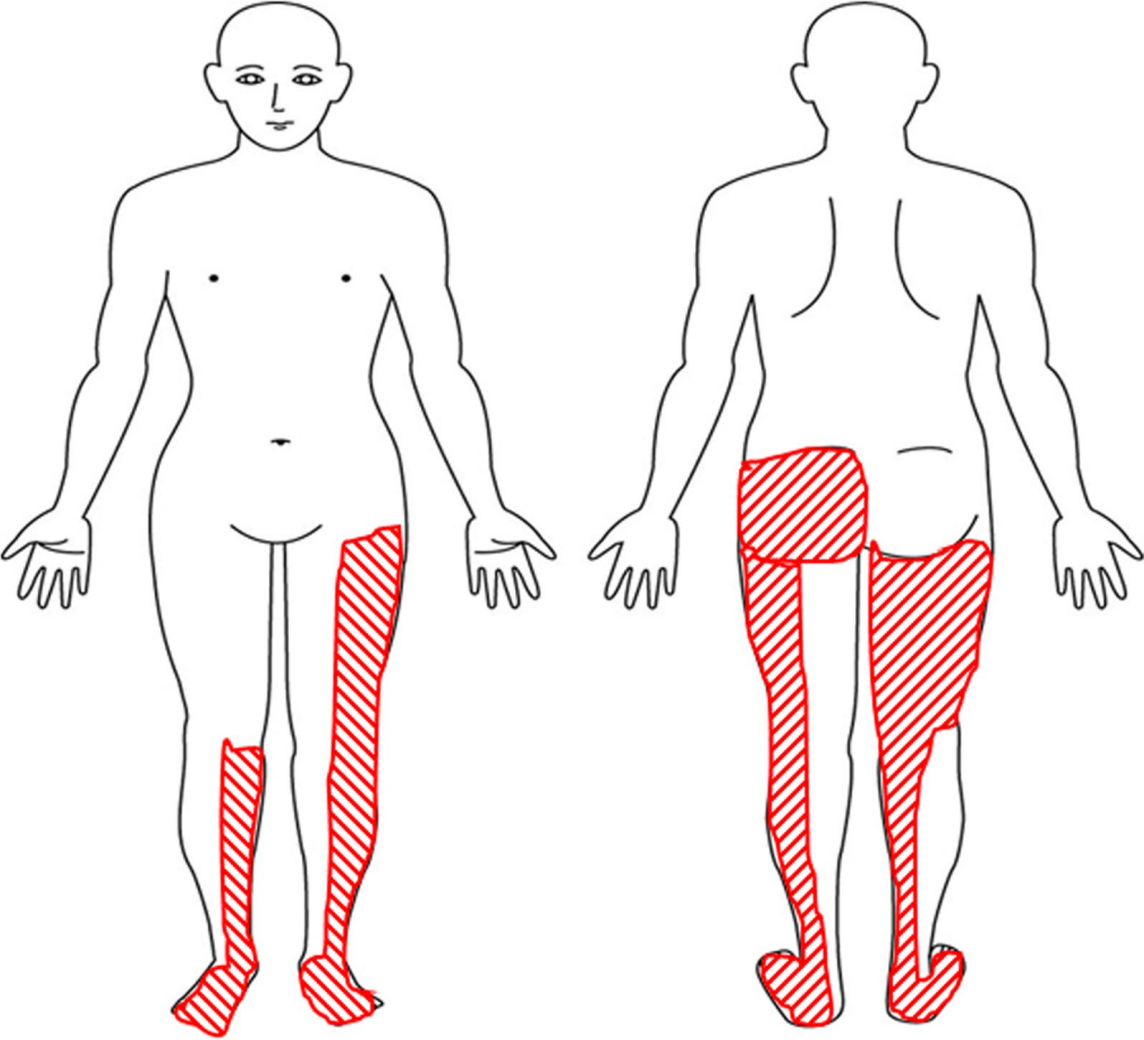
Areas showing dysesthesia (red diagonal)

He was fully conscious, and with pulse rate, temperature, blood pressure, and respiratory rate of 117 beats/min, 36.5 °C, 153/110 mmHg, and 15 breaths/min, respectively. A physical examination did not reveal any rash, or any other obvious abnormal findings while neurological examination was negative for Jolt accentuation, no nuchal rigidity, and Kerning's and Brudzinski's signs. However, the Babinski reflexes wore positive bilaterally, and he had right patellar tendon hyperreflexia and positive Romberg and Mann tests. In addition, he had urinary retention and constipation, and decreased anal sphincter reflex, a reduction in vibratory sensation in both lower limbs and dysesthesia in parts of the lower limbs as illustrated in Fig. 1. There were no other abnormalities neurologic examination. The Laboratory test results on presentation are shown in Table 1. The abnormalities included relative neutrophilia, and elevated levels of D-dimer, procalcitonin, lactic dehydrogenase, blood urea nitrogen and creatinine.

**Table 1:** Laboratory data on admission

Parameter	Recorded value	Standard value
White blood cell count	7920/µL	4500–7500/µL
Neutrophils	81.4%	42–74%
Lymphocytes	13.5%	18–50%
Monocytes	4.7%	1–10%
Hemoglobin	16.8 g/dL	11.3–15.2 g/dL
Platelet count	335 × 10^3^/µL	130–350 × 10^3^/µL
Prothrombin time/international normalized ratio	1.02	0.80–1.20
Activated partial thromboplastin time	27.9 s	26.9–38.1 s
D-dimer	2.1 μg/mL	< 1.0 μg/mL
C-reactive protein	0.03 mg/L	≤ 0.60 mg/dL
Procalcitonin	0.97 ng/mL	≤ 0.05 ng/mL
Total protein	8.0 g/dL	6.9–8.4 g/dL
Albumin	4.4 g/dL	3.9–5.1 g/dL
Total bilirubin	1.2 mg/dL	0.2–1.2 mg/dL
Aspartate aminotransferase	26 U/L	11–30 U/L
Alanine aminotransferase	37 U/L	4–30 U/L
Lactase dehydrogenase	190 U/L	109–216 U/L
Creatine kinase	44 U/L	40–150 U/L
Blood urea nitrogen	35.8 mg/dL	8–20 mg/dL
Creatinine	1.50 mg/dL	0.63–1.03 mg/dL
Sodium	138 mEq/L	136–148 mEq/L
Potassium	4.4 mEq/L	3.6–5.0 mEq/L
Chloride	100 mEq/L	98–108 mEq/L
Glucose	117 mg/dL	70–109 mg/dL
Hemoglobin A1c	5.8%	5.6–5.9%

**Fig. 2:**
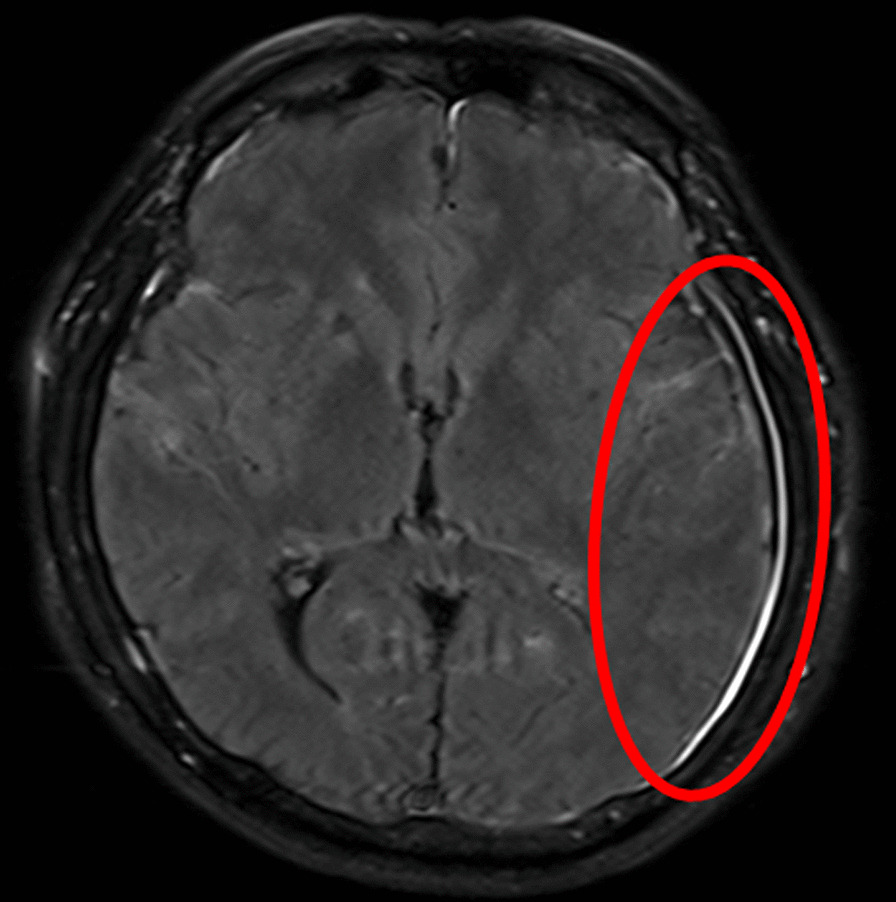
Head contrast-enhanced MRI showing meningeal thickening and contrast enhancement (red circle)

No abnormalities were noted in urinalysis. Abdominal computed tomography (CT) revealed urinary and fecal retentions, but there was no space-occupying lesion in the pelvis nor a lumbar spinal canal stenosis. No abnormalities were observed in the spine on magnetic resonance imaging (MRI), but cranial contrast-enhanced MRI showed meningeal thickening (Fig. 2).

Because the cranial MRI indicated meningitis and MRS was suspected to be the cause of his acute urinary retention, his cerebrospinal fluid was examined for confirmation of the diagnosis. The results were as follows: cell count of 232/μL (mononuclear cells 227/μL, polymorphonuclear cells 5/μL), protein level of 331 mg/dL, sugar level of 69 mg/dL (47.6% of the simultaneous blood glucose level of 145 mg/dL), and adenosine deaminase concentration of 14.5 U/L. Furthermore, the CSF was positive for VZV DNA, but negative for cryptococcal Ag, and general bacterial, and mycobacterium cultures. The titers of various viruses in the serum are shown in Table 2. The elevated VZV IgM level indicated acute phase infection, while the high VZV IgG level indicated a reactivation of VZV. Elevated Epstein-Barr virus viral capsid antigen IgG and Epstein-Barr virus nuclear antigen IgG indicated past Epstein-Barr virus infection, while the negative Epstein-Barr virus IgM ruled-out acute Epstein-Barr virus infection.

**Table 2:** Titers of various viruses in the serum

Parameter	Recorded value	standard value
Human immunodeficiency virus antigen antibody	(−)	(−)
Hepatitis B surface antigen	(−)	(−)
Hepatitis C virus antibody	(−)	(−)
Rapid plasma reagent	(−)	(−)
*Treponema pallidum* latex agglutination	(−)	(−)
EB-VCA IgM	0.0 EIA value	< 0.4 EIA value
EB-VCA IgG	7.4 EIA value	< 0.4 EIA value
EBNA IgG	4.7 EIA value	< 0.4 EIA value
HSV IgM	0.15 EIA value	< 0.7 EIA value
HSV IgG	< 2.0 EIA value	< 2.0 EIA value
VZV IgM	8.24 EIA value	< 0.8 EIA value
VZV IgG	128.0 EIA value	< 2.0 EIA value
Mumps virus IgM	0.04 EIA value	< 0.7 EIA value
Mumps virus IgG	2.6 EIA value	< 2.0 EIA value

*EB-VCA* Epstein-Barr virus viral capsid antigen, *EBNA* EBV Epstein-Barr virus nuclear antigen, *HSV* herpes simplex virus, *VZV* varicella zoster virus, *EIA* enzyme immunometric assay

**Fig. 3:**
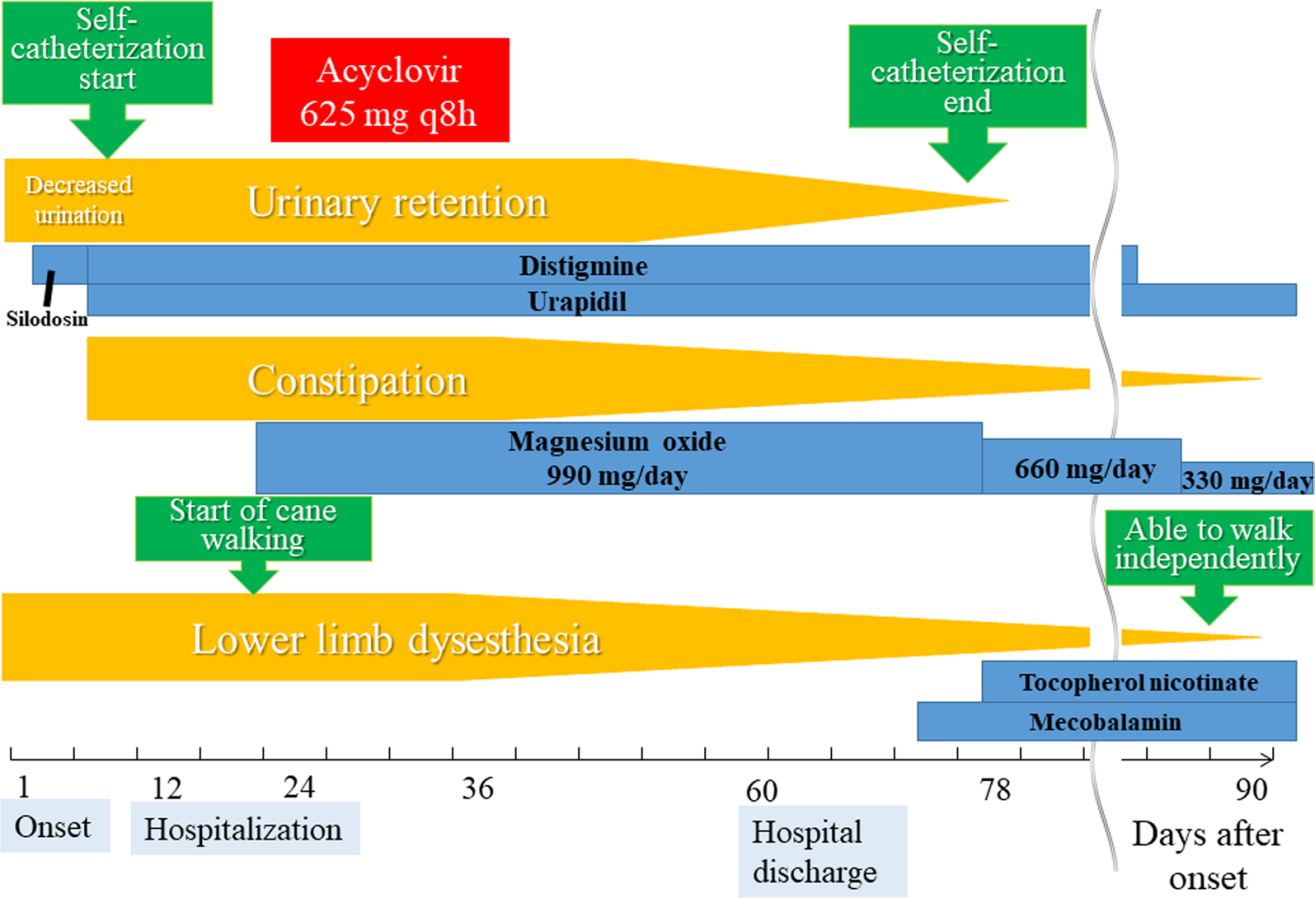
Clinical course of symptoms

The final diagnosis was MRS owing to aseptic meningitis due to VZV. After the diagnosis, he was given intravenous acyclovir at a dose of 625 mg every 8 h for 14 days. The subsequent course is shown in Fig. 3. The symptoms gradually improved, and management with oral medications (distigmine, urapidil, magnesium oxide) became feasible.

The medications for dysuria and dyschezia have been discontinued, and 2 years have passed since the onset of MRS. No sequelae have been observed, and the patient is being followed up.

## Discussion and conclusions

Except for fever, no findings indicative of meningitis were observed on presentation, and diagnosing the condition as VZV meningitis without a rash was difficult.

MRS is a rare condition that presents with complications of aseptic meningitis and dysuria [2]. Furthermore, MRS due to VZV is rare [2, 4, 5, 7, 10–12]. To the best of our knowledge, only one previous case of VZV meningitis without a rash that led to MRS has been reported [5]. The mechanism of MRS is presumed to include spinal shock owing to meningeal irritation, inflammation of upper motor neurons of the spinal cord, direct viral entry, and onset of acute disseminated encephalomyelitis after viral infection [13].

No rash was not observed before and after the onset. A previous study reported that skin findings were not observed in 40% of patients with meningoencephalitis caused by VZV [6]. Thus, the possibility of VZV should be considered in the differential diagnosis of aseptic meningitis, despite the absence of a rash. If urinary retention without an identifiable cause is present, MRS due to aseptic meningitis should be considered in the differential diagnosis, and cerebrospinal fluid should be examined, despite a lack of signs of meningitis.

In conclusion, meningitis due to VZV without a rash is possible, as is meningitis without specific signs and symptoms. Therefore, despite the absence of symptoms or signs suggestive of meningitis, MRS owing to aseptic meningitis, including VZV, should be considered in the differential diagnosis of acute urinary retention.

## Data Availability

All data generated or analyzed during this study are included in this published article.

## Abbreviations

CT: Computed tomography
Ig: Immunoglobulin
MRI: Magnetic resonance imaging
MRS: Meningitis retention syndrome
VZV: Varicella zoster virus
